# Germ-line and somatic *EPHA2* coding variants in lens aging and cataract

**DOI:** 10.1371/journal.pone.0189881

**Published:** 2017-12-21

**Authors:** Thomas M. Bennett, Oussama M’Hamdi, J. Fielding Hejtmancik, Alan Shiels

**Affiliations:** 1 Department of Ophthalmology and Visual Sciences, Washington University School of Medicine, St. Louis, Missouri, United States of America; 2 Ophthalmic Genetics and Visual Function Branch, National Eye Institute, National Institutes of Health, Bethesda, Maryland, United States of America; CNR, ITALY

## Abstract

Rare germ-line mutations in the coding regions of the human EPHA2 gene (*EPHA2*) have been associated with inherited forms of pediatric cataract, whereas, frequent, non-coding, single nucleotide variants (SNVs) have been associated with age-related cataract. Here we sought to determine if germ-line *EPHA2* coding SNVs were associated with age-related cataract in a case-control DNA panel (> 50 years) and if somatic *EPHA2* coding SNVs were associated with lens aging and/or cataract in a post-mortem lens DNA panel (> 48 years). Micro-fluidic PCR amplification followed by targeted amplicon (exon) next-generation (deep) sequencing of *EPHA2* (17-exons) afforded high read-depth coverage (1000x) for > 82% of reads in the cataract case-control panel (161 cases, 64 controls) and > 70% of reads in the post-mortem lens panel (35 clear lens pairs, 22 cataract lens pairs). Novel and reference (known) missense SNVs in *EPHA2* that were predicted *in silico* to be functionally damaging were found in both cases and controls from the age-related cataract panel at variant allele frequencies (VAFs) consistent with germ-line transmission (VAF > 20%). Similarly, both novel and reference missense SNVs in *EPHA2* were found in the post-mortem lens panel at VAFs consistent with a somatic origin (VAF > 3%). The majority of SNVs found in the cataract case-control panel and post-mortem lens panel were transitions and many occurred at di-pyrimidine sites that are susceptible to ultraviolet (UV) radiation induced mutation. These data suggest that novel germ-line (blood) and somatic (lens) coding SNVs in *EPHA2* that are predicted to be functionally deleterious occur in adults over 50 years of age. However, both types of *EPHA2* coding variants were present at comparable levels in individuals with or without age-related cataract making simple genotype-phenotype correlations inconclusive.

## Introduction

Cataract(s) is a clinically heterogeneous disorder that causes clouding or opacification of the crystalline lens and, thereby, impairs refraction and focusing of light onto the photosensitive retina of the eye. Typically, cataract is acquired with aging (> 50 years) and, despite surgical treatment, age-related cataract remains a leading cause of adult visual impairment (17%-33%) and blindness (33%-51%) worldwide [1–3]. Besides aging, epidemiological studies have identified multiple environmental or lifestyle risk factors for age-related cataract including, solar UV-radiation exposure, tobacco smoking, and diabetes [4–6]. In addition, genetic factors are believed to account for 35–58% of the risk for age-related cataract [7,8]. Beyond age-related cataract, congenital, infantile and childhood forms of cataract that occur with relatively low prevalence (1–15 cases/10,000) account for 1.4%-34% of pediatric visual impairment globally [9–12]. Etiological studies of pediatric cataract reveal that genetic causes account for 10%-39% of cases; however, this may represent an underestimate since 50%-60% of cases are deemed idiopathic [10,11,13]. So far, genetic studies have identified at least 30 genes underlying inherited forms of pediatric cataract and several of these genes have also been implicated in the much more common forms of age-related cataract [14,15].

EPH-receptor A2 (EPHA2) is a member of the erythropoietin-producing hepatocellular-carcinoma (EPH) sub-family of receptor tyrosine kinases (RTKs) that play critical signaling roles in embryonic development, adult tissue homeostasis, and cancer development and progression [16–20]. Structurally, EPHA2 is a type-1 (single-pass) transmembrane glycoprotein (~130kDa) with multiple functional domains including an extracellular (N-terminal) ligand binding domain (LBD) for eph-receptor interacting (ephrin) ligands and cytoplasmic (C-terminal) domains including a tyrosine kinase (TK) signaling domain and a sterile-α-motif (SAM) domain implicated in receptor clustering and protein-protein interactions [21,22]. First identified as epithelial cell kinase (eck) [23], EPHA2 is widely expressed in epithelial tissues and is surprisingly abundant in the plasma-membrane proteome of the ocular lens in both humans and mice [24,25], where it is believed to function in lens cell migration and organization [26–29].

Genetic studies have identified germ-line mutations in the human EPHA2 gene (*EPHA2*) on chromosome 1p that underlie inherited forms of pediatric cataract exhibiting both autosomal dominant and recessive modes of inheritance [30–43]. *EPHA2*-related cataract may present at birth (congenital), during infancy or during childhood and displays variable clinical morphology including posterior polar opacities, nuclear opacities, cortical opacities and total lens opacities (https://sites.wustl.edu/catmap). Currently, the *EPHA2* mutation spectrum includes 14 missense mutations predicted to result in amino-acid substitutions, one nonsense mutation, and five frame-shift mutations predicted to result in either C-terminally truncated or extended proteins. Most of these mutations (13/20) occur in cytoplasmic domains of EPHA2 with four mutations clustered within the SAM domain and two in the TK domain. Ectopic overexpression studies in cultured cells suggest that mutations in the SAM domain destabilize the receptor and/or impair targeting to the plasma-membrane [44,45].

Beyond rare mutations, single nucleotide polymorphisms/variants (SNPs/SNVs) across the *EPHA2* region have been variably associated with the much more prevalent forms of age-related cataract including cortical cataract, posterior sub-capsular cataract (PSC) and mixed forms of lens opacities in Caucasian/European, Asian/Indian and Chinese populations [26,30, 46–50]. While most of the associated SNVs were located in non-coding or untranslated regions (UTRs), at least one rare, non-synonymous (missense), coding SNV (rs116506614) predicted to result in an amino-acid substitution (p.R721Q) has been associated with age-related cataract [26]. Further, *in silico* prediction analysis suggests that several other missense SNVs in *EPHA2* (e.g. rs229180, p.E825K) may have deleterious effects on receptor function [51] and expression of several *EPHA2* coding SNVs (rs1058371—p.I96F, p.E825K) in cultured lens epithelial cells has been associated with receptor destabilization and increased susceptibility to oxidative stress [52]. These observations suggest that rare coding SNVs in *EPHA2* may increase susceptibility to age-related forms of cataract. Here we sought to determine whether rare coding SNVs in *EPHA2*, of either germ-line or somatic origin, were associated with lens aging and/or age-related cataract.

## Materials and methods

### Ethics statement

Ethical approval for this study was obtained from the University of Parma, the National Eye Institute, and Washington University (IRB ID #: 201111056 and 00–0320), and written informed consent was provided in accordance with the tenets of the Declaration of Helsinki.

### Cataract case-control DNA panel

Genomic DNA was extracted using standard methods from blood samples donated by a case-control cohort of unrelated individuals age ≥50 years form Northern Italy that were ascertained from the Clinical Trial of Nutritional Supplements (CTNS) and Age-Related Cataract Study [53,54]. Cataract status (nuclear, cortical, posterior sub-capsular, clear lens) was evaluated by grading slit-lamp and retro-illumination lens photographs according to a modification of the Age-Related Eye Disease Study (AREDS) cataract grading system as described [55].

### Lens DNA panel

Post-mortem human donor lenses (≥48 years of age, with or without cataract) were obtained (on dry-ice) from the National Disease Research Interchange (http://ndriresource.org/). Lens genomic DNA was extracted using the DNeasy Kit (Qiagen, Valencia, CA) essentially according to the manufacturer’s protocol with the following modifications to mitigate the high protein-to-DNA content of the lens. Each lens was homogenized (2 min—setting 8, Bullet Blender 24, Next Advance, Averill park, NY) in buffer ATL (360 ul) then digested (16 hr, 56°C) with proteinase K (40 ul 15 mg/ml). Samples were then diluted with buffer ATL (360ul) and re-digested (2 hr, 56°C) with proteinase K (40 ul) followed by centrifugation (5 min, 10,000 x g) to remove excess protein before processing through spin-columns according to the manufacturer’s instructions. DNA was eluted from the spin-columns in buffer AE (200 ul) and quantified (OD_260_) using a spectrophotometer (ND-2000, NanoDrop, Wilmington, DE). If necessary, samples were concentrated by air-drying in a laminar-flow hood and re-suspended in ultrapure water to give a minimum concentration of 50 ng/μl required for amplicon sequencing.

### Targeted-amplicon deep-sequencing and variant calling

Targeted-amplicon deep-sequencing was performed using the Access Array Integrated Fluidic Circuit (IFC) System with custom designed and validated gene-specific adaptor-primers (Fluidigm, San Francisco, CA). Each IFC enables nanoliter-volume high-throughput PCR to generate amplicons (≤200 bp) across 48 samples in a single run for subsequent next-generation (deep)-sequencing (NGS). Briefly, DNA samples (50 ng) and primers were mixed ‘on-chip’ (48.48 Access Array IFC/pre-PCR IFC Controller AX), and PCR amplified (FC1 Cycler). Amplicons for each sample were pooled on-chip (post-PCR IFC Controller AX) then indexed with sample barcodes and NGS adaptors (Access Array Barcode kit) to produce 48 sequencer-ready libraries. Sequencing-by-synthesis was performed on the MiSeq platform (Illumina, San Diego, CA). Paired-end reads were aligned to the human reference genome (hg19) with Novoalign (www.novocraft.com) and processed using the Sequence Alignment/Map (SAM) tools software package and Picard programs (http://samtools.sourceforge.net/). Variants were called using the FreeBayes program (https://arxiv.org/abs/1207.3907) for germ-line variants and the VarScan 2 program for somatic variants [56,57] (http://varscan.sourceforge.net). Finally, selected SNVs were confirmed in both directions by manual inspection using the Integrative Genomics Viewer (IGV) browser [58] (http://software.broadinstitute.org/software/igv/).

### Statistical analysis

Genetic association analysis and logistic regression analysis of selected SNVs found in the cataract case-control panel was performed using the Golden Helix SNP and Variation Suite 7 (Golden Helix, Bozeman, MT). Statistical comparison of somatic SNVs found in the post-mortem lens panel was performed using Fisher’s Exact Test by means of the online spreadsheet at http://www.langsrud.com/fisher.htm. A probability (*p*) value of < 0.05 after correction for multiple testing was considered significant.

## Results

### DNA panels

The cataract case-control panel comprised 225 leukocyte DNA samples from 161 patients with age-related cataract (age 50+) and 64 age-matched clear lens controls from the N. Italian population [53,54]. The cataract cases included 67 nuclear only, 43 cortical only, and two posterior sub-capsular cataract (PSC) only. In addition to ‘pure’ forms of cataract, there were multiple cases of mixed cataract including 21 nuclear + cortical, 14 nuclear + PSC, 10 cortical + PSC, and four nuclear + cortical + PSC. The mean age of cataract cases = 74.2 ± SD 6.54 years (range 50–85 years) and the mean age of clear lens controls = 75.19 ± SD 4.2 years (range 57–86), with no significant difference between cases and controls (*p* = 0.21). The sex distribution was 50% female and 50% male in the cases and 44% female and 56% male in the controls. There was no association between any cataract and sex in the case-control panel using chi-square test (*p* = 0.51).

Post-mortem donor lenses were briefly examined at the time of procurement for the presence or absence of obvious age-related cataract prior to cryopreservation. However, the donor information report did not identify age-related cataract sub-types (e.g. nuclear, cortical). Further, we cannot exclude the possibility that cataract in some of these donor lenses may have been associated with causes other than aging (e.g. uveitis). The post-mortem lens panel comprised 118 genomic DNA samples extracted from 74 clear lenses (37 pairs) and 44 cataract lenses (22 pairs) all obtained from Caucasian donors (age 48+ years). Two of the clear lens pairs failed amplicon sequencing and/or QC criteria leaving 114 lens samples (35 clear pairs, 22 cataract pairs) for variant analysis. The mean age of cataract lenses = 65.5 ± SD 6.67 (range 48–74 years) and the mean age of clear lenses = 64.06 ± SD 7.37 (range 48–78 years) with no significant difference between the two groups (*p* = 0.45). The sex distribution was 23% female and 77% male in the cataract lenses and 49% female and 51% male in the clear lenses. Despite the numerical sex difference in the cataract lenses there was no significant association between any cataract and sex in the post-mortem lens panel using chi-square test (*p* = 0.095).

### Targeted-amplicon deep-sequencing of exonic variants

We performed targeted-amplicon deep-sequencing of the coding regions (exons) of the human EPHA2 gene to identify germ-line single nucleotide variants (SNVs) in the cataract case-control panel and somatic SNVs in the lens panel. *EPHA2* (GeneID: 1969) spans ~31.8 Kbp on the short (p) arm of chromosome 1 (cytogenetic band region 1p36.1) [59] with a physical location between nucleotides (nt) 16124337–16156104 (counted from the short-arm telomere, ptel) on the complement strand [Annotation release 108, Genome Reference Consortium Human Build 38 patch release 7 (GRCh38.p7)] (http://www.ncbi.nlm.nih.gov/gene/1969). Currently, the gene reference sequence (NG_021396.1) comprises 17 coding exons generating two transcript variants, NM_004431.4 and NM_001329090.1, encoding protein isoforms of 976 amino acids (NP_004422.2) and 922 amino acids (NP_001316019), respectively. For comparison with *EPHA2*, we simultaneously performed amplicon sequencing of the gene coding for cellular tumor antigen p53 (*TP53*)—a tumor suppressor gene that is known to acquire somatic mutations in several cancers (e.g. cutaneous melanoma) (http://cancer.sanger.ac.uk/cosmic). *TP53* (Gene ID: 7157) spans ~19.15 Kbp on chromosome 17p13.1 (7668402–7687550, complement) and the gene reference sequence (NG_017013.2) comprises 11 coding exons giving rise to 8 transcript variants and 12 protein isoforms (a-l) ranging from 182–393 amino-acids (http://www.ncbi.nlm.nih.gov/gene/7157).

Optimal custom design of PCR primer pairs (Fluidigm) to amplify exons for deep-sequencing resulted in 35 amplicons for *EPHA2* and 15 amplicons for *TP53*. Across the cataract case-control panel the mean total number of reads was 418,214 with > 99% on target of which > 82% attained 1000x coverage (S1 Table). Similarly, across the lens panel the mean total number of reads was 456,286 with > 99% on target of which > 70% attained 1000x coverage (S1 Table). All amplicons were fully sequenced in both directions with the exception of amplicon 35 in *EPHA2* (part of exon-1) likely due to its high G/C content.

Following sequencing, germ-line SNVs in the cataract case-control panel (blood leukocyte DNA) were called using the FreeBayes program. Variant allele frequencies (VAFs) were calculated as a percentage by dividing the number of individual variant reads by the total number of amplicon reads and those SNVs with VAFs ≥ 20% were designated germ-line. Somatic variants in the lens DNA panel were called using the VarScan 2 program that was originally designed to call low-frequency (> 1%) somatic variants from deep-sequencing data derived from matched tumor (case) versus control tissue samples [56,57]. For our purposes, we compared left and right lenses from the same individual using the paired analysis or somatic mode. Rare variants present in both lenses were designated as germ-line, whereas, those present in only the left or the right lens (i.e. discordant SNVs) were designated as somatic. In order to reduce the risk of false positives we excluded somatic SNVs with VAFs below 3% and/or coverage depths below 600 reads as potential sequencing errors. For convenience, germ-line and somatic SNVs were divided into novel and reference categories to denote their absence or presence, respectively, in public genome databases including the Single Nucleotide Polymorphism database (dbSNP build 138), Exome Variant Server (EVS), Exome Aggregation Consortium (ExAC), 1000 Genomes project (1000G), and Catalogue of Somatic Mutations in Cancer (COSMIC). Both categories predominantly contained synonymous and non-synonymous (i.e. missense) SNVs with *in silico* predictions of damaging or deleterious effects at the protein level determined using appropriate algorithms (e.g. SIFT and PolyPhen-2). Binary versions (.bam files) of the Sequence Alignment/Map (.sam) files have been deposited with the NIH Short Read Archive (SRA Accession no. PRJNA384802).

### Germ-line *EPHA2* variants in the cataract case-control panel

Exon deep-sequencing of *EPHA2* in the cataract case-control panel detected 10 novel SNVs (all transitions) and 20 reference SNVs (18 transitions) in the exon regions of *EPHA2* at VAFs >20%—consistent with germ-line transmission (Table 1). Of the novel SNVs, two were synonymous and eight were non-synonymous—predicted to result in missense amino-acid substitutions. Two of the novel missense SNVs (p.I142T, p.W348R) occurred in controls and both were predicted *in silico* to be damaging. Of the remaining six missense SNVs found in cases, two were predicted *in silico* to be benign (p.A650T, p.A932T) and four damaging (p.G171E, p.G776S, p.N831D, p.L895P). Since nine of the novel SNVs occurred only once in the panel, and the other only twice, we were unable to perform further statistical analysis.

**Table 1:** Germ-line *EPHA2* coding SNVs found in the cataract case-control panel (VAF >20%).

Exon	Ref. Seq. no.	DNA Change	Amino-Acid Change	Protein Domain	PolyPhen-2 (prediction)	SIFT (prediction)	MAF—EVS Caucasian	MAF—1000G (CEU)	MAF—Cataract Case-Control Panel	Clear lens	Cortical Cataract	Nuclear Cataract	PSC	Mixed Cataract
2	rs147977279	c.121C>G	p.L41V	LBD	0.998 (D)	0.00 (D)	0.0003	0.0000	0.0022	0	1	0	0	0
3	Novel	c.425T>C	p.I142T	LBD	0.959 (D)	0.00 (D)			0.0022	1	0	0	0	0
3	Novel	c.512G>A	p.G171E	LBD	0.883 (P)	0.00 (D)			0.0022	0	1	0	0	0
3	rs147352564	c.523C>T	p.R175C	LBD	0.985 (D)	0.00 (D)	0.0012	0.0000	0.0022	0	1	0	0	0
3	rs6678618	c.570G>A	p.A190A	LBD	synonymous	synonymous	0.3455	0.3586	0.3800	38	27	43	1	32
3	rs6678616	c.573G>A	p.L191L	LBD	synonymous	synonymous	0.3457	0.3586	0.3600	38	26	40	1	28
3	rs34753465	c.648C>T	p.A216A		synonymous	synonymous	0.0053	0.0152	0.0089	0	1	2	0	1
4	Novel	c.852G>A	p.E284E		synonymous	synonymous			0.0022	0	0	0	0	1
5	rs2230597	c.987C>T	p.P329P		synonymous	synonymous	0.4032	0.4444	0.4311	41	30	44	1	35
5	Novel	c.1042T>C	p.W348R		1.000 (D)	0.01 (D)			0.0022	1	0	0	0	0
5	rs374687482	c.1089C>T	p.S363S		synonymous	synonymous	0.0000	0.0000	0.0022	0	0	1	0	0
5	rs34192549	c.1171G>A	p.G391R		0.001 (B)	0.11 (T)	0.0173	0.0101	0.0067	1	1	1	0	1
5	rs55700006	c.1314G>A	p.E438E		synonymous	synonymous	0.0028	0.0000	0.0067	0	0	2	1	0
6	rs55740291	c.1359C>T	p.S453S		synonymous	synonymous	0.0001	0.0000	0.0067	1	0	2	0	0
11	rs55655135	c.1896G>A	p.L632L	TK	synonymous	synonymous	0.0081	0.0101	0.0133	1	2	3	0	0
11	Novel	c.1948G>A	p.A650T	TK	0.433 (B)	0.22 (T)			0.0022	0	0	0	0	1
11	rs10907223	c.1983C>T	p.L661L	TK	synonymous	synonymous	0.0381	0.0404	0.0556	9	6	6	0	4
11	Novel	c.2016 C>T	p.H672H	TK	synonymous	synonymous			0.0022	0	0	1	0	0
13	rs116506614	c.2162G>A	p.R721Q	TK	1.000 (D)	0.000 (D)	0.0012	0.0051	0.0044	1	1	0	0	0
13	rs145592908	c.2239G>A	p.V747I	TK	0.999 (D)	0.000 (D)	0.0005	0.0000	0.0044	0	1	1	0	0
14	Novel	c.2326G>A	p.G776S	TK	1.000 (D)	0.00 (D)			0.0022	0	0	1	0	0
14	rs112285834	c.2352C>T	p.T784T	TK	synonymous	synonymous	0.0160	0.0152	0.0489	6	5	2	0	6
15	Novel	c.2491A>G	p.N831D	TK	0.937 (D)	0.00 (D)			0.0044	0	0	0	0	2
15	rs35903225	c.2627G>A	p.R876H		0.999 (D)	0.000 (D)	0.0256	0.0202	0.0089	2	1	0	0	1
15	rs142789236	c.2669G>A	p.R890H		0.999 (D)	0.000 (D)	0.0005		0.0067	2	0	0	0	1
16	Novel	c.2684T>C	p.L895P		1.000 (D)	0.00 (D)			0.0022	0	0	0	0	1
16	Novel	c.2794G>A	p.A932T	SAM	0.000 (B)	1.00 (T)			0.0022	0	0	1	0	0
17	rs3754334	c.2874C>T	p.I958I	SAM	synonymous	synonymous	0.2813	0.3131	0.3200	34	25	37	1	25
17	rs138818894	c.2904G>C	p.Q968H	SAM	0.031 (B)	1.000 (T)	0.0035	0.0000	0.0044	1	0	0	0	1
17	rs114895977	c.2919G>A	p.G973G		synonymous	synonymous	0.0015	0.0000	0.0044	1	0	1	0	0

(B)—benign, (P)—probably damaging, (D)—damaging, (T)—tolerated

Of the reference SNVs, 12 were synonymous and eight were predicted to result in missense amino-acid substitutions (Table 1). Of the eight missense reference SNVs six were predicted to result in damaging amino-acid substitutions—with two occurring in cases only (p.L41V, p.R175C) and three occurring in both cases and controls (p.R721Q, p.R876H, p.R890H). The minor allele frequencies (MAFs) for all reference SNVs found in the cataract case-control panel were similar to those reported in Caucasians by public genome variant databases (Table 1). Four of the synonymous reference SNVs that were relatively common in the Caucasian population (MAF 28%-44%) were also the most common in the cataract case-control panel (S2a Table). However, only one of these SNVs (rs6678616) showed weak association (*p* = 0.032) with nuclear cataract and nuclear cataract + PSC using Fisher’s Exact Test (S2b Table). Correcting for sex using logistic regression in the association analysis of rs6678616 did not provide significant association with any type of cataract (*p* > 0.24). The remainder of synonymous reference SNVs occurred in cases and/or controls but were comparatively rare in the panel (MAF < 1%) hampering further statistical analysis.

### Germ-line *TP53* variants in the cataract-case control panel

Exon deep-sequencing of *TP53* in the cataract case-control panel detected no novel SNVs and only nine reference SNVs (5 transitions) of which five were also present in the COSMIC database (Table 2 and S3a Table). Two of these SNVs (rs1042522, rs730882008) were non-synonymous and predicted *in silico* (SIFT) to be damaging, with one (rs1042522, p.P72R) present at relatively high frequency in Caucasians (MAF 0.25) and in multiple cases and controls. However, rs1042522 was not associated with any type of cataract (p > 0.33) using Fisher’s Exact Test (S3b Table). Correcting for sex with logistic regression in the association analysis of rs1042522 did not provide significant association with any type of cataract (*p* = 0.85). The other SNV (rs730882008, p.R282L) occurred at unknown frequency in the population and in only one case of cortical cataract preventing further statistical analysis.

**Table 2:** Germ-line *TP53* coding SNVs found in the cataract case-control panel (VAF >20%).

Exon	Ref. Seq. No.	COSMIC ID No.	DNA change	Amino Acid Change	PolyPhen-2 (prediction)	SIFT (prediction)	MAF-EVS Caucasian	MAF—1000G (CEU)	MAF—Cataract Case-Control Panel	Clear Lens	Cortical Cataract	Nuclear Cataract	PSC	Mixed Cataract
4	rs1800370		c.108G>A	p.P36P	synonymous	synonymous	0.0148	0.0051	0.0178	2	1	4		1
4	rs1042522	COSM250061	c.215C>G	p.P72R	0.083 (B)	0.03 (D)	0.2548*	0.2424*	0.2666*	60	38	64	2	46
4	rs751978853		c.354A>T	p.T118T	synonymous	synonymous			0.0022	1				
5	rs375275361	COSM45823	c.558T>C	p.D186D	synonymous	synonymous			0.0022		1			
6	rs1800372	COSM249885	c.639A>G	p.R213R	synonymous	synonymous	0.0193	0.0202	0.0333	7		5		3
8	rs770598448		c.789T>C	p.N263N	synonymous	synonymous			0.0022					1
8	rs730882008	COSM44470	c.845G>T	p.R282L	0.998 (D)	0.00 (D)			0.0022		1			
8	rs200073907	COSM45332	c.885T>C	p.P295P	synonymous	synonymous			0.0044					2
11	rs765530090		c.1113C>A	p.S371S	synonymous	synonymous			0.1800	24	17	26	1	13

*MAF refers to reference C allele. (B)—benign, (D)—damaging

### Somatic *EPHA2* variants in the post-mortem lens panel

Exon deep-sequencing of *EPHA2* in the lens panel detected a total of 935 discordant SNVs (VAF > 1%) in 35 pairs of clear lenses and 726 discordant SNVs in 22 pairs of cataract lenses suggesting a somatic origin (S4 and S5 Tables). We arbitrarily selected a VAF cut-off threshold value of ≥ 3% to minimize false-positive sequencing errors. In the clear lenses, 109 discordant SNVs occurred with a VAF of ≥ 3% in 27 of the 35 clear lens pairs; however, 43 were excluded due to low coverage (read-depth <600). The remaining 66 SNVs, each of which occurred only once in the clear lens pairs, included 28 synonymous SNVs, 32 non-synonymous or missense SNVs resulting in missense substitutions, 3 stop-gain or nonsense SNVs, 2 UTR SNVs, and one splice-site SNV (Table 3). Of these SNVs, only 14 were listed in reference databases (e.g. snp138, cosmic70, exac01) suggesting that 52 were novel somatic SNVs. Of the 32 missense SNVs, only nine were listed in reference databases (e.g. snp138, cosmic70) and 29 were predicted *in silico* (by the SIFT algorithm) to be damaging (Table 3). Surprisingly, 31 of the 32 missense SNVs involved C/T or G/A transitions and 17 of these occurred at di-pyrimidine sites that are susceptible to UV-induced mutation [60]. Similarly, 18 of the 28 synonymous SNVs along with two UTR SNVs and two nonsense SNVs occurred at UV-susceptible di-pyrimidine sites (Table 3).

**Table 3:** Somatic *EPHA2* coding SNVs found in the paired clear lens panel (VAF >3%).

Chr	Start/End	Ref	Alt	ExonicFunc.refGene	AAChange.refGene	cosmic70	snp138	exac01	SIFT	Depth	VAF
chr1	16451690	G*	A*	UTR-3	NM_004431:c.*20C>T			1.58E-05		3502	3.94%
chr1	16451707	G*	A*	UTR-3	NM_004431:c.*3C>T					4181	3.28%
chr1	16451720	A	G	nonsynonymous SNV	EPHA2:NM_004431:exon17:c.T2921C:p.I974T				D	4172	3.96%
chr1	16451809	G*	A*	synonymous SNV	EPHA2:NM_004431:exon17:c.C2832T:p.I944I					1416	4.31%
chr1	16451815	G	A	synonymous SNV	EPHA2:NM_004431:exon17:c.C2826T:p.D942D		rs143828420	8.69E-05		1417	4.10%
chr1	16455972	C*	T*	nonsynonymous SNV	EPHA2:NM_004431:exon16:c.G2782A:p.A928T				D	3242	12.94%
chr1	16456009	G*	A*	synonymous SNV	EPHA2:NM_004431:exon16:c.C2745T:p.S915S					7158	13.87%
chr1	16456014	C	A	stopgain	EPHA2:NM_004431:exon16:c.G2740T:p.E914X	ID = COSM3934228			D	3242	8.34%
chr1	16456016	A	G	nonsynonymous SNV	EPHA2:NM_004431:exon16:c.T2738C:p.L913P				D	3048	3.28%
chr1	16456023	C*	T*	nonsynonymous SNV	EPHA2:NM_004431:exon16:c.G2731A:p.E911K		rs376030072		D	3991	4.44%
chr1	16456039	G*	A*	synonymous SNV	EPHA2:NM_004431:exon16:c.C2715T:p.P905P					3055	5.24%
chr1	16456067	G*	A*	nonsynonymous SNV	EPHA2:NM_004431:exon16:c.C2687T:p.P896L				T	2950	5.29%
chr1	16456744	C*	T*	synonymous SNV	EPHA2:NM_004431:exon15:c.G2646A:p.K882K					4205	4.04%
chr1	16456822	C	A	nonsynonymous SNV	EPHA2:NM_004431:exon15:c.G2568T:p.Q856H				D	4309	7.80%
chr1	16458240	G*	A*	synonymous SNV	EPHA2:NM_004431:exon14:c.C2451T:p.P817P					6056	3.32%
chr1	16458257	T	C	nonsynonymous SNV	EPHA2:NM_004431:exon14:c.A2434G:p.T812A				D	5718	3.06%
chr1	16458352	G*	A*	nonsynonymous SNV	EPHA2:NM_004431:exon14:c.C2339T:p.P780L				D	1668	8.69%
chr1	16458353	G*	A*	nonsynonymous SNV	EPHA2:NM_004431:exon14:c.C2338T:p.P780S				D	1668	5.28%
chr1	16458579	C*	T*	nonsynonymous SNV	EPHA2:NM_004431:exon13:c.G2305A:p.E769K		rs367724183	2.37E-05	D	1247	6.52%
chr1	16458598	G	A	synonymous SNV	EPHA2:NM_004431:exon13:c.C2286T:p.R762R					2564	33.35%
chr1	16458890	G*	A*	synonymous SNV	EPHA2:NM_004431:exon12:c.C2098T:p.L700L					1776	3.72%
chr1	16458893	C*	T*	nonsynonymous SNV	EPHA2:NM_004431:exon12:c.G2095A:p.A699T	ID = COSM1727288			D	1780	4.22%
chr1	16458896	C*	T*	nonsynonymous SNV	EPHA2:NM_004431:exon12:c.G2092A:p.G698R				D	1781	4.27%
chr1	16458911	C*	T*	nonsynonymous SNV	EPHA2:NM_004431:exon12:c.G2077A:p.E693K				D	1776	4.23%
chr1	16458927	G*	A*	synonymous SNV	EPHA2:NM_004431:exon12:c.C2061T:p.P687P					1775	3.90%
chr1	16459729	T	C	nonsynonymous SNV	EPHA2:NM_004431:exon11:c.A1999G:p.M667V				D	2940	8.80%
chr1	16459977	T	C	synonymous SNV	EPHA2:NM_004431:exon10:c.A1863G:p.A621A					3560	3.65%
chr1	16460030	A	G	nonsynonymous SNV	EPHA2:NM_004431:exon10:c.T1810C:p.F604L				D	2125	4.33%
chr1	16460049	G*	A*	synonymous SNV	EPHA2:NM_004431:exon10:c.C1791T:p.P597P					3711	3.18%
chr1	16460050	G*	A*	nonsynonymous SNV	EPHA2:NM_004431:exon10:c.C1790T:p.P597L				D	3700	3.22%
chr1	16460066	G*	A*	nonsynonymous SNV	EPHA2:NM_004431:exon10:c.C1774T:p.H592Y				D	3736	3.28%
chr1	16460068	G*	A*	nonsynonymous SNV	EPHA2:NM_004431:exon10:c.C1772T:p.P591L				D	3486	3.30%
chr1	16460401	G*	A*	synonymous SNV	EPHA2:NM_004431:exon9:c.C1692T:p.N564N					1197	7.20%
chr1	16460407	C*	T*	synonymous SNV	EPHA2:NM_004431:exon9:c.G1686A:p.R562R					2464	20.06%
chr1	16460962	C*	T*	splicing	NM_004431:exon9:c.1682+1G>A					4208	5.28%
chr1	16461003	G*	A*	nonsynonymous SNV	EPHA2:NM_004431:exon8:c.C1642T:p.L548F				D	1415	3.67%
chr1	16461007	G*	A*	synonymous SNV	EPHA2:NM_004431:exon8:c.C1638T:p.V546V					3799	5.00%
chr1	16461024	C	T	nonsynonymous SNV	EPHA2:NM_004431:exon8:c.G1621A:p.V541M		rs61731097	2.26E-03	D	3766	3.58%
chr1	16462157	C	T	nonsynonymous SNV	EPHA2:NM_004431:exon6:c.G1421A:p.R474H				D	4070	3.02%
chr1	16464354	G*	A*	stopgain	EPHA2:NM_004431:exon5:c.C1306T:p.Q436X				D	4515	3.43%
chr1	16464480	G*	A*	nonsynonymous SNV	EPHA2:NM_004431:exon5:c.C1180T:p.R394C			2.37E-05	D	4989	5.46%
chr1	16464490	G	A	synonymous SNV	EPHA2:NM_004431:exon5:c.C1170T:p.H390H		rs113173342	1.02E-03		4613	4.10%
chr1	16464498	G*	A*	nonsynonymous SNV	EPHA2:NM_004431:exon5:c.C1162T:p.P388S				D	4640	5.07%
chr1	16464513	C	T	nonsynonymous SNV	EPHA2:NM_004431:exon5:c.G1147A:p.V383M	ID = COSM1205441			D	7947	3.60%
chr1	16464529	C*	T*	synonymous SNV	EPHA2:NM_004431:exon5:c.G1131A:p.G377G					2588	3.21%
chr1	16464550	C*	T*	stopgain	EPHA2:NM_004431:exon5:c.G1110A:p.W370X				T	7850	3.80%
chr1	16464553	G*	A*	synonymous SNV	EPHA2:NM_004431:exon5:c.C1107T:p.C369C					2488	12.42%
chr1	16464583	G	A	synonymous SNV	EPHA2:NM_004431:exon5:c.C1077T:p.D359D					5120	3.03%
chr1	16464596	C*	T*	nonsynonymous SNV	EPHA2:NM_004431:exon5:c.G1064A:p.G355E				D	2489	3.50%
chr1	16464607	A	G	synonymous SNV	EPHA2:NM_004431:exon5:c.T1053C:p.P351P					1090	3.13%
chr1	16464618	A	G	nonsynonymous SNV	EPHA2:NM_004431:exon5:c.T1042C:p.W348R				D	1808	3.98%
chr1	16464621	G	A	nonsynonymous SNV	EPHA2:NM_004431:exon5:c.C1039T:p.R347C			7.90E-06	D	1442	3.81%
chr1	16464625	C*	T*	synonymous SNV	EPHA2:NM_004431:exon5:c.G1035A:p.E345E					1659	18.26%
chr1	16464641	A	G	nonsynonymous SNV	EPHA2:NM_004431:exon5:c.T1019C:p.M340T				D	1011	4.95%
chr1	16464655	G*	A*	synonymous SNV	EPHA2:NM_004431:exon5:c.C1005T:p.L335L					2505	4.47%
chr1	16464658	G*	A*	synonymous SNV	EPHA2:NM_004431:exon5:c.C1002T:p.Y334Y					2078	8.81%
chr1	16464664	T	C	synonymous SNV	EPHA2:NM_004431:exon5:c.A996G:p.P332P					2070	3.54%
chr1	16464822	A	G	synonymous SNV	EPHA2:NM_004431:exon4:c.T927C:p.C309C					6671	3.36%
chr1	16475091	G*	A*	nonsynonymous SNV	EPHA2:NM_004431:exon3:c.C605T:p.P202L				D	3906	4.74%
chr1	16475108	G*	A*	synonymous SNV	EPHA2:NM_004431:exon3:c.C588T:p.V196V					3928	3.39%
chr1	16475144	A	G	synonymous SNV	EPHA2:NM_004431:exon3:c.T552C:p.D184D					2676	10.31%
chr1	16475374	A	G	nonsynonymous SNV	EPHA2:NM_004431:exon3:c.T322C:p.F108L		rs149867517	7.89E-06	T	5817	4.06%
chr1	16475408	A	G	synonymous SNV	EPHA2:NM_004431:exon3:c.T288C:p.I96I					4013	12.52%
chr1	16475446	T	C	nonsynonymous SNV	EPHA2:NM_004431:exon3:c.A250G:p.N84D				T	5681	3.15%
chr1	16475451	C	T	nonsynonymous SNV	EPHA2:NM_004431:exon3:c.G245A:p.R82H			1.58E-05	D	5662	3.55%
chr1	16477406	G*	A*	synonymous SNV	EPHA2:NM_004431:exon2:c.C138T:p.H46H			7.89E-06		4868	4.36%

*SNV at di-pyrimidine site. D—damaging, T—tolerated.

In the cataract lenses, 35 discordant *EPHA2* SNVs occurred with a VAF ≥ 3% in 10 of the 22 cataract lens pairs with only two excluded due to low read-depth (S5 Table). The remaining 33 singly occurring SNVs included 12 synonymous SNVs, 19 non-synonymous or missense SNVs, and two stop-gain or nonsense SNVs (Table 4). Of these SNVs, six were present in reference databases suggesting that 27 were novel somatic SNVs and only one (at position 16460407 bp) was present in both cataract and clear lenses (Tables 3 and 4). Of the 19 missense SNVs only four were present in reference databases and 15 were predicted *in silico* (SIFT) to be damaging (Table 4). All 19 missense SNVs involved C/T or A/G transitions and 12 of these occurred at UV-susceptible di-pyrimidine sites. Ten of the 12 synonymous SNVs and both nonsense SNVs also occurred at UV-susceptible di-pyrimidine sites. Overall for *EPHA2*, there was no significant difference between the paired clear lens panel and the paired cataract lens panel with respect to total SNVs (*p* = 0.48), damaging SNVs (*p* = 0.85), or novel SNVs (*p* = 0.64) using Fisher’s Exact Test (S6 Table). Correcting for sex in the lens panels using logistic regression analysis did not provide any significant association for total *EPHA2* SNVs (*p* = 0.62), damaging *EPHA2* SNVs (*p* = 0.63), or novel *EPHA2* SNVs (*p* = 0.70).

**Table 4:** Somatic *EPHA2* coding SNVs found in the paired cataract lens panel (VAF >3%).

Chr	Start/End	Ref	Alt	ExonicFunc.refGene	AAChange.refGene	cosmic70	snp138	exac01	SIFT	Depth	VAF
chr1	16456045	C*	T*	synonymous SNV	EPHA2:NM_004431:exon16:c.G2709A:p.G903G			1.58E-05		3596	3.81%
chr1	16456068	G*	A*	nonsynonymous SNV	EPHA2:NM_004431:exon16:c.C2686T:p.P896S				T	2885	5.03%
chr1	16456083	C	T	nonsynonymous SNV	EPHA2:NM_004431:exon16:c.G2671A:p.V891M		rs139168333	7.11E-05	T	3220	3.63%
chr1	16456749	G*	A*	nonsynonymous SNV	EPHA2:NM_004431:exon15:c.C2641T:p.L881F				D	3694	6.37%
chr1	16456804	G*	A*	synonymous SNV	EPHA2:NM_004431:exon15:c.C2586T:p.P862P					5679	4.19%
chr1	16456871	A	G	nonsynonymous SNV	EPHA2:NM_004431:exon15:c.T2519C:p.M840T				D	1851	3.08%
chr1	16458249	G	A	synonymous SNV	EPHA2:NM_004431:exon14:c.C2442T:p.G814G					5698	8.80%
chr1	16458309	G*	A*	synonymous SNV	EPHA2:NM_004431:exon14:c.C2382T:p.F794F					3319	6.03%
chr1	16458692	G*	A*	nonsynonymous SNV	EPHA2:NM_004431:exon13:c.C2192T:p.A731V				D	3257	4.49%
chr1	16458703	C*	T*	nonsynonymous SNV	EPHA2:NM_004431:exon13:c.G2181A:p.M727I				D	3645	4.61%
chr1	16458763	C*	T*	synonymous SNV	EPHA2:NM_004431:exon13:c.G2121A:p.K707K					635	3.46%
chr1	16459847	C	T	synonymous SNV	EPHA2:NM_004431:exon11:c.G1881A:p.V627V					2735	4.64%
chr1	16460407	C*	T*	synonymous SNV	EPHA2:NM_004431:exon9:c.G1686A:p.R562R					3157	4.25%
chr1	16462261	G*	A*	synonymous SNV	EPHA2:NM_004431:exon6:c.C1317T:p.P439P					2620	3.32%
chr1	16464353	T	C	nonsynonymous SNV	EPHA2:NM_004431:exon5:c.A1307G:p.Q436R				D	3943	3.61%
chr1	16464600	T	C	nonsynonymous SNV	EPHA2:NM_004431:exon5:c.A1060G:p.S354G				D	2232	6.14%
chr1	16464608	G*	A*	nonsynonymous SNV	EPHA2:NM_004431:exon5:c.C1052T:p.P351L				D	1669	4.38%
chr1	16464609	G*	A*	nonsynonymous SNV	EPHA2:NM_004431:exon5:c.C1051T:p.P351S				D	2969	4.65%
chr1	16464610	G*	A*	synonymous SNV	EPHA2:NM_004431:exon5:c.C1050T:p.P350P					3004	4.23%
chr1	16464614	G	A	nonsynonymous SNV	EPHA2:NM_004431:exon5:c.C1046T:p.T349M		rs200490325	2.37E-04	D	3066	4.08%
chr1	16464617	C*	T*	stopgain	EPHA2:NM_004431:exon5:c.G1043A:p.W348X				T	2426	6.84%
chr1	16464623	A	G	nonsynonymous SNV	EPHA2:NM_004431:exon5:c.T1037C:p.L346P			7.90E-06	D	2780	3.42%
chr1	16464624	G*	A*	synonymous SNV	EPHA2:NM_004431:exon5:c.C1036T:p.L346L					1668	4.62%
chr1	16464633	T	C	nonsynonymous SNV	EPHA2:NM_004431:exon5:c.A1027G:p.K343E				D	2716	3.57%
chr1	16464665	G*	A*	nonsynonymous SNV	EPHA2:NM_004431:exon5:c.C995T:p.P332L				D	1708	4.64%
chr1	16464666	G*	A*	nonsynonymous SNV	EPHA2:NM_004431:exon5:c.C994T:p.P332S				D	1698	4.71%
chr1	16464790	G*	A*	nonsynonymous SNV	EPHA2:NM_004431:exon4:c.C959T:p.P320L				T	6624	3.30%
chr1	16464917	G*	A*	nonsynonymous SNV	EPHA2:NM_004431:exon4:c.C832T:p.P278S	ID = COSM1185338			T	602	3.49%
chr1	16474897	C*	T*	nonsynonymous SNV	EPHA2:NM_004431:exon3:c.G799A:p.E267K				D	2893	6.22%
chr1	16475162	G*	A*	synonymous SNV	EPHA2:NM_004431:exon3:c.C534T:p.F178F					6441	3.46%
chr1	16475177	G*	A*	synonymous SNV	EPHA2:NM_004431:exon3:c.C519T:p.L173L			2.37E-05		5784	3.46%
chr1	16475269	C*	T*	nonsynonymous SNV	EPHA2:NM_004431:exon3:c.G427A:p.D143N				D	3573	8.28%
chr1	16475541	C*	T*	stopgain	EPHA2:NM_004431:exon3:c.G155A:p.W52X				T	3510	7.67%

*SNV at di-pyrimidine site. D—damaging, T—tolerated.

### Somatic *TP53* variants in the post-mortem lens panel

Exon deep-sequencing of *TP53* in the lens panel detected a total of 392 discordant SNVs (VAF > 1%) in 35 clear lens pairs and 298 discordant SNVs in 22 cataract lens pairs (S7 and S8 Tables). In the clear lenses, 64 discordant SNVs were present at a VAF > 3% in 27 of the 35 pairs; however, 12 of these SNVs were excluded due to low read-depth (<600). In addition, three discordant SNVs occurred more than once—one non-synonymous SNV (COSM1658764) occurred in nine lenses, one synonymous SNV (present in ExAC01) occurred in 11 lenses, and one UTR SNV occurred in two lenses—resulting in a total of 19 SNVs that were excluded for recurrence. The remaining 33 single occurrence SNVs included nine synonymous SNVs (8 transitions), 16 non-synonymous or missense SNVs (15 transitions), seven UTR SNVs (all transitions), and one splicing SNV (transition). Of these SNVs, 18 were present in reference databases (e.g. cosmic, snp138, exac01) leaving 15 putatively novel somatic SNVs (S7d Table). Of the 16 missense SNVs, 13 were present in reference databases, seven were predicted *in silico* (SIFT) to be damaging and five occurred at UV-susceptible di-pyrimidine sites. Apart from the splicing SNV, none of the synonymous SNVs or UTR SNVs occurred at di-pyrimidine sites (S7d Table).

In the cataract lenses, 18 discordant *TP53* SNVs (all transitions) occurred with VAFs > 3% in five of the 22 pairs of lenses including five synonymous SNVs, 12 non-synonymous or missense SNVs, and one UTR-3’ SNV (S8d Table). Of these single occurrence SNVs, 12 were present in reference databases leaving six potentially novel somatic SNVs and only one (at position 7572892 bp) was present in both cataract and clear lenses (S7d and S8d Tables). Of the 12 missense SNVs, eight were present in reference databases, six were predicted to be damaging, and 11 occurred, along with the UTR SNV, at UV-susceptible di-pyrimidine sites (S8d Table). Overall for *TP53*, there was no significant difference between the paired clear lens panel and the paired cataract lens panel with respect to total SNVs (*p* = 0.73), damaging SNVs (*p* = 0.77), or novel SNVs (*p* = 0.78) using Fisher’s Exact Test (S9 Table). Correcting for sex in the lens panels using logistic regression analysis did not provide any significant association for total TP53 SNVs (*p* = 0.39), damaging TP53 SNVs (*p* = 0.71), or novel TP53 SNVs (*p* = 0.57).

## Discussion

In this study we utilized targeted-amplicon (exon) deep-sequencing to identify germ-line and somatic variants of *EPHA2*—particularly novel missense variants predicted *in silico* to result in deleterious amino-acid substitutions—that may be associated with lens aging and/or age-related cataract. First, we profiled germ-line SNVs (VAF > 20%) in *EPHA2* for association with age-related cataract in a Caucasian case-control panel that had previously revealed association with common reference SNVs flanking *EPHA2* [30]. Exon deep-sequencing detected six novel missense SNVs and eight reference missense SNVs in the cataract case-control panel that were predicted to be damaging (Table 1). However, the relatively small number of individuals in the cataract case-control panel that harbored these damaging *EPHA2* SNVs (n < 20) limited the power of this study to detect disease association. For example, of two novel SNVs located in the extracellular LBD of EPHA2 one (p.I142T) was present in a control, while the other (p.G171E) occurred in a case with cortical cataract. Similarly, one of the reference missense SNVs, rs116506614 (c.2162G>A, p.R721Q), located in the TK domain of EPHA2, that has previously been associated with age-related cortical cataract [26], was present in a case with cortical cataract and in a control from our cataract case-control panel. Overall, while it is possible that such control individuals may be pre-symptomatic for age-related cataract, we note that other putatively deleterious SNVs were found only in controls, whereas, putatively benign SNVs were present in cases (Table 1) rendering simple genotype-phenotype correlations inconclusive.

Second, we profiled putative somatic SNVs in *EPHA2* (VAF ≥ 3%) that arose in post-mortem lenses procured from Caucasian donors over 48 years of age (Tables 3 and 4). Paired analysis of right and left lenses from the same individual for discordant SNVs, analogous to that of matched tumor versus control tissues, detected 19 novel missense SNVs in a clear lens panel (35 pairs) and 13 novel missense SNVs in a cataract lens panel (22 pairs) that were predicted to be damaging (Tables 3 and 4). By comparison, the same paired-lens analysis of *TP53* for discordant SNVs yielded predominantly reference somatic SNVs found in the COSMIC database and no novel SNVs that were predicted to be damaging (S7 and S8 Tables). This difference in SNV profile between the two genes likely reflects the high frequency of somatic mutations identified in *TP53* versus *EPHA2*. Currently, the COSMIC database lists over 29,480 somatic mutations in *TP53* including 17,166 missense substitutions that have been detected in multiple tumor samples (e.g. cutaneous melanoma) at relatively high frequencies (~27%). By contrast, *EPHA2* harbors some 275 somatic mutations including 164 missense substitutions that have been detected in multiple tumor samples (e.g. stomach, intestine, skin), at relatively low frequencies (typically < 5%) (http://cancer.sanger.ac.uk/cosmic). These observations suggest that novel somatic variants in *EPHA2* that are predicted to be functionally deleterious are detectable in aging human lenses. Overall, our data are in agreement with a recent study that employed targeted-hybridization deep-sequencing of human lens epithelial samples to identify somatic variants in a panel of 151 cancer-related genes [61]. To the best of our knowledge, this is the first report of putative somatic mutations in a lens-expressed gene causally implicated in age-related cataract. However, since rudimentary statistical analysis confirmed that somatic SNVs in *EPHA2* were present at comparable frequencies in both clear lenses and those with age-related cataract we are unable to determine if such variants are causative for disease.

A striking feature of both the germ-line and the somatic missense SNVs in *EPHA2* detected here was the high frequency of transitions (C/T, G/A) versus transversions (G/C, G/T, A/C, A/T). Theoretically, transversions should occur twice as often as transitions; however, a review of the germ-line variation annotated in the *EPHA2* reference sequence reveals that the vast majority of missense variants involve C/T or G/A transitions (http://www.ncbi.nlm.nih.gov/variation/view/). The occurrence of somatic C>T transitions is of particular interest since they may result from exposure to solar UV radiation [60]. Absorption of solar UV radiation (95% UV-A, 5% UV-B) by DNA promotes the formation of photodimeric lesions, mostly cyclobutane pyrimidine dimers (CPDs), at adjacent pyrimidine bases (C and T) that may escape nucleotide excision repair leading to base substitution and generation of UV-signature mutations (C>T or CC>TT) during DNA replication [62]. Among the somatic missense SNVs detected in our lens panel (clear and cataract) many of the C>T changes (G>A on the complementary strand) were present at di-pyrimidine (diPy) sites (CT, TC, CC) in both *EPHA2* and *TP53* raising the possibility that they represent UV-signature mutations (Tables 3 and 4 and S7 and S8 Tables). While there was no significant association between these somatic SNVs and cataract in our lens panel, epidemiological studies have established that lifetime exposure to solar UV radiation (particularly UV-B) is a significant risk factor for cortical cataract particularly within the lens nasal quadrant [63,64]. In addition, UV-A radiation has been implicated in the increased prevalence of left-sided cortical cataract and facial skin cancer, likely in part, due to increased exposure while operating left-hand drive vehicles [65]. Further, it has been suggested that oxidative stress secondary to solar UV exposure might contribute to age-related cataract [66]. However, since the cornea effectively absorbs most solar UV-B radiation (290–320 nm) and the levels of CPDs in lens epithelia obtained from cataract patients has been reported to be relatively low compared to those of oxidized purines, the cause-effect relationship between solar UV exposure and age-related cataract remains unclear [67,68]. Future studies of somatic variants, including UV-signature mutations, in *EPHA2* and over 30 other known cataract genes, including those for crystallins (e.g. CRYAA), connexins (e.g. *GJA8*) and ocular transcription factors (e.g. *HSF4*) [14,15] may provide new insights regarding the molecular genetic mechanisms underlying age-related cataract.

## Supporting information

S1 Table Amplicon deep-sequencing coverage in the cataract case-control panel (a) and the post-mortem lens panel (b). (XLSX)

S2 Table Germ-line *EPHA2* coding SNV frequency (a) and association (b) in the cataract case-control panel (VAF >20%). (XLSX)

S3 Table Germ-line *TP53* coding SNV frequency (a) and association (b) in the cataract case-control panel (VAF >20%). (XLSX)

S4 Table Somatic *EPHA2* coding SNVs found in the paired clear lens panel. (XLSX)

S5 Table Somatic *EPHA2* coding SNVs found in the paired cataract lens panel. (XLSX)

S6 Table Fisher’s exact test of *EPHA2* coding SNVs found in the post-mortem lens panel. (XLSX)

S7 Table Somatic *TP53* coding SNVs found in the paired clear lens panel. (XLSX)

S8 Table Somatic *TP53* coding SNVs found in the paired cataract lens panel. (XLSX)

S9 Table Fisher’s exact test of *TP53* coding SNVs found in the post-mortem lens panel. (XLSX)
